# Inhibition of the production of mediators of inflammation by corticosteroids is a glucocorticoid receptor-mediated process

**DOI:** 10.1155/S0962935196000166

**Published:** 1996-04

**Authors:** G. S. Madretsma, A. P. M. van Dijk, C. J. A. M. Tak, J. H. P. Wilson, F. J. Zijlstra

**Affiliations:** 1 Department of Pharmacology Faculty of Medicine and Health Sciences Erasmus University Rotterdam The Netherlands; 2 Department of Internal Medicine II University Hospital Dijkzigt Rotterdam The Netherlands

## Abstract

In order to find an explanation for corticosteroid resistance we assessed whether inhibition by dexamethasone (DEX) of the stimulated production of TNF-∝, IL-6, PGE_2_ and LTB_4_ by peripheral blood mononuclear cells (MNC) depends on binding to the glucocorticoid receptor (GR), and whether it is determined by the number or the affinity of the GR of these cells. GR number and affinity of MNC were determined by means of a whole cell DEX binding assay. MNC were incubated with DEX and LPS or A23187 in the absence or presence of RU486, a potent steroid antagonist. DEX caused a concentration dependent inhibition of TNF-∝, IL-6 and PGE_2_ production but had no effect on LTB_4_ production. RU486 significantly blocked the effect of DEX, but no correlations were found between the inhibition of mediator release and the K_d_ or receptor number.


Mediators of Inflammation 5, 100–103 (1996)

### Inhibition of the production of mediators of inflammation by corticosteroids is a glucocorticoid receptor-mediated process

G. S. Madretsma,[1,2,CA] A. P. M. van Dijk,[1,2] C. J. A. M. Tak,[1] J. H. P. Wilson[2] and F. J. Zijlstra[1]

[1]Department of Pharmacology, Faculty of Medicine and Health Sciences, Erasmus University Rotterdam, The Netherlands. Fax: (+31) 10 4366839; [2]Department of Internal Medicine II, University Hospital Dijkzigt, Rotterdam, The Netherlands

[CA]Corresponding Author

**In order to find an explanation for corticosteroid resistance we assessed whether inhibition by dexamethasone (DEX) of the stimulated production of TNF-α, IL-6, $PGE_2$ and $LTB_4$ by peripheral blood mononuclear cells (MNC) depends on binding to the glucocorticoid receptor (GR), and whether it is determined by the number or the affinity of the GR of these cells. GR number and affinity of MNC were determined by means of a whole cell DEX binding assay. MNC were incubated with DEX and LPS or A23187 in the absence or presence of RU486, a potent steroid antagonist. DEX caused a concentration dependent inhibition of TNF-α, IL-6 and $PGE_2$ production but had no effect on $LTB_4$ production. RU486 significantly blocked the effect of DEX, but no correlations were found between the inhibition of mediator release and the $K_d$ or receptor number.**

**Key words**: Glucocorticoid receptor, inhibition, mediators of inflammation

### Introduction

Corticosteroids decrease the severity of inflammation and cause a subjective improvement in the majority, but not in all, patients with non-infectious inflammatory disorders such as inflammatory bowel disease (IBD),[1] asthma[2] and rheumatoid arthritis.[3] The anti-inflammatory effects of corticosteroids are ascribed to inhibition of the production of mediators of inflammation, including eicosanoids and cytokines.[4–6]

Several control studies have shown that topical or systemic corticosteroids are much more effective than placebo controls, but that not all patients improve. Depending on the dose and route of application, duration of the treatment, severity of the exacerbation and the parameters under investigation, approximately 10–40% of patients do not respond or respond insufficiently to corticosteroids.[7,8]

Stimulation of mononuclear cells (MNC) by lipopolysaccharide (LPS) or Ca-ionophore enhances the production of a range of inflammatory mediators by these cells such as the cytokines tumour necrosis factor-α (TNF-α) and interleukin 6 (IL-6), and the eicosanoids prostaglandin $E_2$ ($PGE_2$) and leukotriene $B_4$ ($LTB_4$).[9] This provides an *in vitro* model for studying the anti-inflammatory properties of corticosteroids.

In order to clarify some aspects of the mechanism through which corticosteroids exert their anti-inflammatory effects we examined the effect of dexamethasone (DEX) on the production of mediators of inflammation by human MNC *in vitro* and assessed whether this effect depends on binding of corticosteroids to the glucocorticoid receptor (GR).

The hypothesis of this study was that the inhibition of the production of mediators of inflammation by corticosteroids is a glucocorticoid receptor-mediated process. We furthermore tested whether the ability of DEX to inhibit the LPS-stimulated production of TNF-α, IL-6, $PGE_2$ and the $Ca^{2+}$-ionophore enhanced $LTB_4$ production by MNC is determined by the number or affinity of glucocorticoid receptors (GR) on these cells.

### Materials and Methods

*Healthy volunteers:* Approval for this study was obtained from the Medical Ethics Committee of the University Hospital Rotterdam. Venous blood was obtained from nine healthy adults, ranging in age from 22 to 37 years, who joined voluntarily after full explanation of the nature, significance and scope of the study. None of the subjects had taken corticosteroids or any other anti-inflammatory drug for a period of at least 4 weeks prior to donating blood.

*Isolation of human MNC:* Mononuclear cells were isolated from the heparinized venous blood immediately after blood sampling. The method used was a modification of the technique originally described by Boyum.[10] Briefly, the

© 1996 Rapid Science Publishers blood was diluted 1:1 with phosphate buffered saline (PBS; Oxoid, UK) before fractionating it by a one-step Ficoll–Paque gradient (Pharmacia, Sweden) centrifugation at 1100 × **g** for 15 min at 20°C. The interphase was washed in PBS and resuspended in Dulbecco's Modified Eagle's Medium (DMEM) containing HEPES and foetal calf serum (Gibco, UK), supplemented with penicillin and streptomycin (Flow Lab, UK). Cells were stained by Hemacolor (Merck, Germany), the final yield of MNC was greater than 95% and the cell viability (tested by Trypan blue exclusion) was over 97%.

*Culturing of MNC and measurement of cytokines and eicosanoids:* The cells (2 × $10^6$ per well) were cultured in DMEM and incubated in the presence or absence of varying concentrations of DEX (Genfarma, The Netherlands) for 24 h prior to stimulation with LPS (5 μg/ml, *E. coli* 0111:B4, Sigma, USA) for 24 h or $Ca^{2+}$-ionophore (A23187, 1 μM, Sigma, USA) for 15 min. All incubations were performed at 37°C in a humidified atmosphere of 5% $CO_2$ and 95% air. DEX was dissolved in culture medium, and the concentration of DEX varied from $10^{-9}$ to $10^{-5}$ M.

TNF-α and IL-6 were measured by commercially available ELISA kits (Central Laboratory for Blood Transfusion, The Netherlands), whereas $PGE_2$ and $LTB_4$ were determined by specific radioimmunoassays (standards, Sigma, USA; $^3$H-labels, Amersham, UK; and antibodies, Advanced Magnetics Inc., USA).

The effects of different concentrations of DEX on secretion of TNF-α, IL-6 and $PGE_2$ were expressed as percentage inhibition of production in control cultures (cells incubated with DMEM plus supplements and LPS).

*Assessment of glucocorticoid receptor number by a whole cell assay:* The method used was that described by Lamberts *et al.*,[11] but a 1000-fold excess of unlabelled RU486 (Roussel, France), a corticoid receptor antagonist, was used instead of an excess of unlabelled DEX. A stock solution was made by dissolving RU486 in ethanol. This solution was diluted in culture medium. The final concentration of ethanol in the assay samples was less than 0.1%.

In order to enhance the dissociation of endogenously bound corticoids, the MNC used in the assay were washed three times in DMEM, each time allowing for an equilibration period of 30 min at 37°C in a shaking waterbath.[12]

Specific binding was determined by subtracting the amount of nonspecifically bound radioligand from the total amount bound. Data were analysed by constructing a Scatchard plot.[13]

*Statistics:* The effect of DEX on the stimulated release of each mediator was studied in nine separate experiments, with blood from nine different donors. Values are expressed as mean ± S.E.M. Control and DEX-treated cell cultures were compared by paired *t*-test. *p*-values $<$ 0.05 were considered significant. The effect of DEX alone versus the effect of DEX after preincubation with RU486 was compared by Manova.

### Results

*Mediator production by MNC:* After the incubation period LPS-stimulated MNC released the mediators measured in the following concentrations: TNF-α, 90 ± 25 pg/$10^6$ cells; IL-6, 1165 ± 320 pg/$10^6$ cells; $PGE_2$, 1420 ± 340 pg/$10^6$. MNC stimulated with $Ca^{2+}$-ionophore released 2970 ± 940 pg $LTB_4$/$10^6$ cells.

*Effect of DEX on mediator release by MNC:* The inhibitory effect of DEX on TNF-α production was of comparable magnitude to that of IL-6 and $PGE_2$, with an $IC_{50}$ value (concentration of DEX that causes a 50% decrease in mediator release) of 65 nM in comparison to 145 and 140 nM for IL-6 and $PGE_2$ respectively. There was a considerable inter-individual variation in $IC_{50}$ values in response to DEX with a 95% confident interval of 10–400 nM for TNF-α, 15–1095 nM for IL-6 and 30–690 nM for $PGE_2$. The effects of DEX on the production of the studied mediators are shown in Fig. 1. DEX had no effect on $Ca^{2+}$ ionophore-stimulated $LTB_4$ release.

*Antagonism of the effects of DEX in MNC by RU486:* Incubation of MNC with RU486, a steroid receptor antagonist, for 2 h prior to the addition of DEX diminished the inhibitory effect of the glucocorticoid in a dose dependent manner (Fig. 2).

*Assessment of glucocorticoid receptor number and $K_d$ in MNC:* Glucocorticoid receptor content was 4430 ± 340 sites/cell (mean ± S.E.M.) and the $K_d$ 9.5 ± 0.7 nM. In Fig. 3 a representative Scatchard plot from one experiment of the binding of $^3$H-DEX to glucocorticoid receptors of MNC is shown. The slope is equal to the negative reciprocal of the dissociation constant ($-1/K_d$), and the intercept on the abscissa equal to the total concentration of receptors ($B_{max}$). No correlations were found between the inhibition of mediator release and $K_d$ or receptor number.

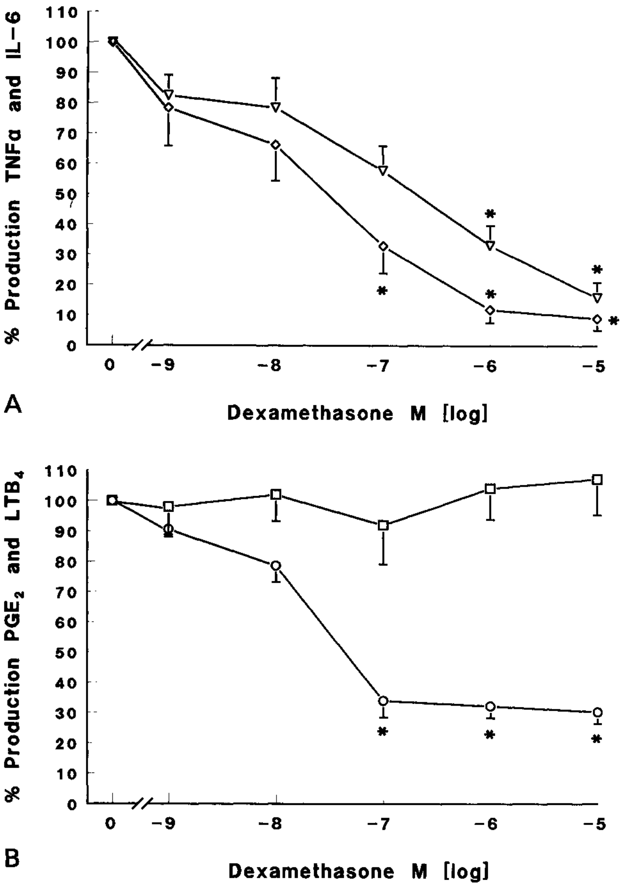

FIG. 1. Effect of DEX on the secretion of (A) TNF-α (◇) and IL-6(▽); and (B) $PGE_2$ (○) and $LTB_4$ (□) by MNC cultures stimulated by LPS (5 μg/ml). Production is presented as a percentage, mean ± S.E.M. (vertical bars), of LPS- or $Ca^{2+}$-ionophore enhanced release and represents nine experiments performed on MNC from nine different donors. *Significance with $p < 0.05$ compared to LPS-enhanced production.

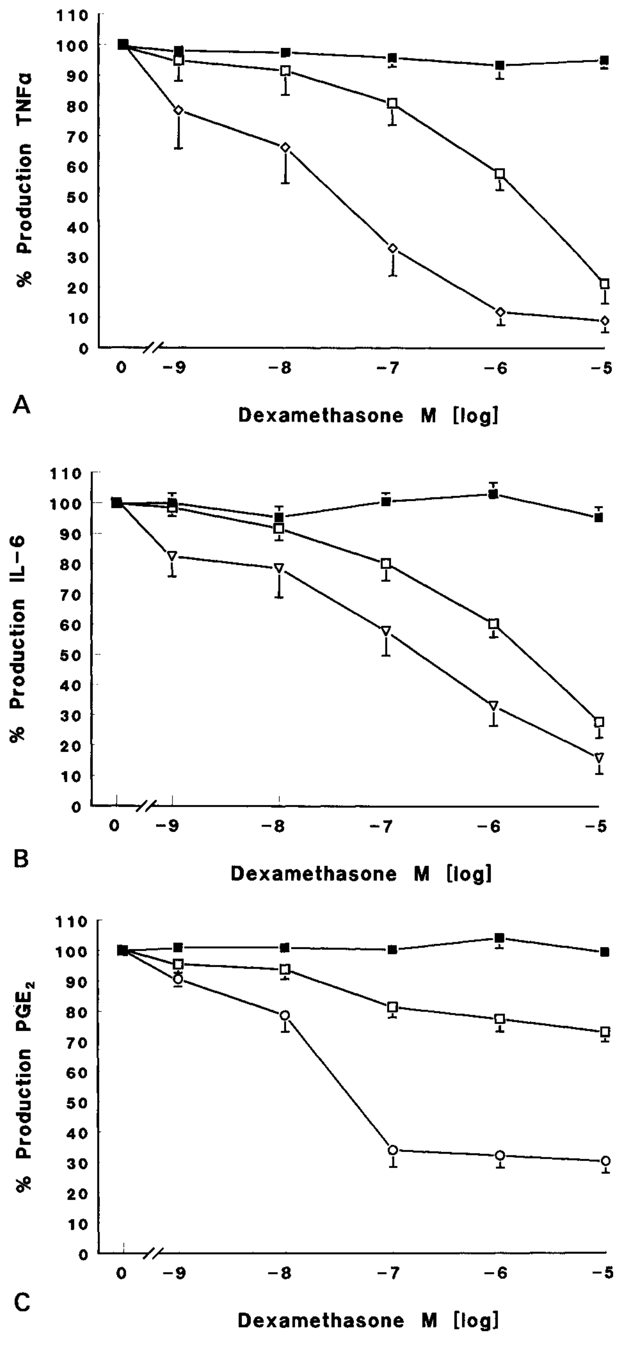

FIG. 2. Effect of DEX on the secretion of (A) TNF-α (◇), (B) IL-6 (▽) and (C) $PGE_2$ (○); and the effect of DEX after preincubation of MNC with RU486 $10^{-8}$ M (□) or $10^{-6}$ M (■). RU486 significantly inhibits the effect of DEX in a dose dependent fashion, $p < 0.001$.

### Discussion

The inhibitory effect of DEX in this *in vitro* experiment varied considerably from donor to donor. The effect of DEX on the release of TNF-α, IL-6 and $PGE_2$ cannot be explained by the effect of DEX on the viability of the cells, otherwise the same effects should have been found for $LTB_4$ release. Instead, no significant differences were found between $LTB_4$ production in the presence versus the absence of DEX. This might indicate that corticosteroids do not inhibit 5-lipoxygenase in MNC as they do the gene expression of cytokine-induced cycloxygenase 2 in monocytes.[14] Furthermore, culture of MNC with DEX, in the concentrations used in this study, did not affect the viability of these cells as determined by trypan blue exclusion. The fact that the effect of DEX could be diminished or even abolished by pre-incubation of the MNC with RU486, a steroid receptor antagonist, strongly suggests a glucocorticoid receptor mediated process.

Mononuclear cells play a central role in chronic inflammation. They possess the capacity to produce eicosanoids and cytokines which modulate the inflammatory response.[15–17] Corticosteroids exert their anti-inflammatory effects partly by inhibiting the production of inflammatory mediators, through a glucocorticoid receptor-mediated process. This means that the GR

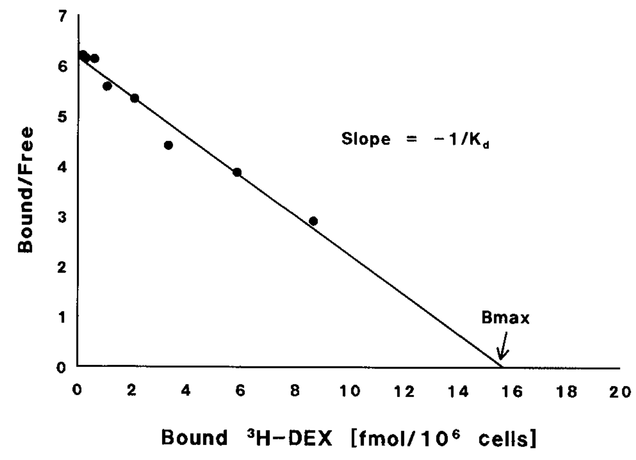

FIG. 3. Scatchard plot of specifically bound $^3$H-dexamethasone to MNC. MNC were incubated with 0–16 nM $^3$H-dexamethasone in the absence or presence of a 1000-fold excess of unlabelled RU486. $K_d$ and $B_{max}$ in the case shown are 12.8 nM and 15.7 fmol/$10^6$ MNC respectively.

plays a crucial role in the anti-inflammatory effects of corticosteroids.

Considering these findings it seems logical that inter-individual variations in reaction to corticosteroids could be explained by variation in the number of GR on the MNC or the affinity of these receptors for DEX. No correlations were found, however, between the $IC_{50}$ of the mediators under investigation and the receptor number or $K_d$. Thus, the degree of response of MNC of healthy donors to DEX does not seem to be determined by the characteristics of the GR, and other factors must play a role. This finding, however, does not exclude the possibility that changes in GR number or affinity are important in glucocorticoid resistance in inflammatory diseases.

The MNC used in this study were isolated from blood obtained from healthy volunteers, who presumably have a normal response to corticosteroids. It is to be expected that comparison of MNC obtained from patients who have proven to be non-responders to corticosteroids, with MNC of responders may reveal differences in GR characteristics more clearly. Studies with MNC isolated from blood obtained from inflammatory bowel disease patients who respond to corticosteroids and patients who do not respond have been initiated.

### References

1. Lennard-Jones JE. Toward optimal use of corticosteroids in ulcerative colitis and Crohn's disease. *Gut* 1983; **24**: 177–181.
2. Kerrebijn KF, van Essen-Zandvliet EEM, Niejens HJ. Effect of long-term treatment with inhaled corticosteroids and beta-agonists on the bronchial responsiveness in children with asthma. *J Allergy Clin Immunol* 1987; **79**: 653–659.
3. Byron MA, Mowat AG. Corticosteroid prescribing in rheumatoid arthritis—the fiction and the fact. *Br J Rheumatol* 1985; **24**: 164–166.
4. Guyre PM, Girard MT, Morganelli PM, Manganiello PD. Glucocorticoid effects on the production and actions of immune cytokines. *J Steroid Biochem* 1988; **30**: 89–93.
5. Schumert R, Towner J, Zipser RD. Role of eicosanoids in human and experimental colitis. *Dig Dis Sci* 1988; **33**: 58S–64S.
6. Dunlap NE, Fulmer JD. Corticosteroid therapy in asthma. *Clin Chest Med* 1984; **5**: 669–683.
7. Lichtiger S, Present DH, Kornbluth A, *et al.* Cyclosporine in severe ulcerative colitis refractory to steroid therapy. *N Engl J Med* 1994; **330**: 1841–1845.
8. Munkholm P, Langholz E, Davidsen M, Binder V. Frequency of glucocorticoid resistance and dependency in Crohn's disease. *Gut* 1994; **35**: 360–362.
9. Pruimboom WM, van Dijk APM, Tak CJAM, Garelds I, Bonta IL, Wilson JHP, Zijlstra FJ. Interactions between cytokines and eicosanoids: a study using human peritoneal macrophages. *Immunol Lett* 1994; **41**: 255–260.
10. Boyum A. Isolation of mononuclear cells and granulocytes from human blood. *Scand J Clin Lab Invest* 1968; **21**: 77–85.
11. Lamberts SWJ, Koper JW, Biemond P, Den Holder FH, De Jong FH. Cortisol receptor resistance: the variability of its clinical presentation and response to treatment. *J Clin Endocrinol Metab* 1992; **74**: 313–321.
12. Shipman GF, Bloomfield CD, Gajl-Peczalska KJ, Munck AU, Smith KA. Glucocorticoids and lymphocytes: effects of glucocorticoid administration on lymphocyte glucocorticoid receptors. *Blood* 1983; **61**: 1086–1090.
13. Scatchard G. The attractions of proteins for small molecules and ions. *Ann N Y Acad Sci* 1949; **51**: 660–672.
14. O'Banion MK, Winn VD, Young DA. cDNA cloning and functional activity of a glucocorticoid-regulated inflammatory cyclooxygenase. *Proc Natl Acad Sci USA* 1992; **89**: 4888–4892.
15. Smith WL. Prostanoid biosynthesis and mechanism of action. *Am J Physiol* 1992; **263**: F181–F191.
16. Lewis RA, Austen KF. The biological active leukotrienes. Biosynthesis, functions and pharmacology. *J Clin Invest* 1984; **73**: 889–897.
17. Vilcek J. Immunology of cytokines: an introduction. In: Thomson A, ed. *The Cytokine Handbook.* London: Academic Press, 1987; 1–17.

ACKNOWLEDGEMENTS. This research project was supported financially by the Netherlands Digestive Diseases Foundation (Nederlandse Lever Darm Stichting). We are much in debt to Dr D. Fekkes. Section Patho-physiology of Behaviour and Dr J. W. Koper, Department of Internal Medicine III, for their advice on the dexamethasone binding assay.

**Received 29 November 1995; accepted in revised form 20 December 1995**

## References

[PDF_00339] Byron M. A., Mowat A. G. (1985). Corticosteroid prescribing in rheumatoid arthritis--the fiction and the fact.. Br J Rheumatol.

[PDF_00357] Böyum A. (1968). Isolation of mononuclear cells and granulocytes from human blood. Isolation of monuclear cells by one centrifugation, and of granulocytes by combining centrifugation and sedimentation at 1 g.. Scand J Clin Lab Invest Suppl.

[PDF_00346] Dunlap N. E., Fulmer J. D. (1984). Corticosteroid therapy in asthma.. Clin Chest Med.

[PDF_00341] Guyre P. M., Girard M. T., Morganelli P. M., Manganiello P. D. (1988). Glucocorticoid effects on the production and actions of immune cytokines.. J Steroid Biochem.

[PDF_00335] Kerrebijn K. F., van Essen-Zandvliet E. E., Neijens H. J. (1987). Effect of long-term treatment with inhaled corticosteroids and beta-agonists on the bronchial responsiveness in children with asthma.. J Allergy Clin Immunol.

[PDF_00359] Lamberts S. W., Koper J. W., Biemond P., den Holder F. H., de Jong F. H. (1992). Cortisol receptor resistance: the variability of its clinical presentation and response to treatment.. J Clin Endocrinol Metab.

[PDF_00333] Lennard-Jones J. E. (1983). Toward optimal use of corticosteroids in ulcerative colitis and Crohn's disease.. Gut.

[PDF_00372] Lewis R. A., Austen K. F. (1984). The biologically active leukotrienes. Biosynthesis, metabolism, receptors, functions, and pharmacology.. J Clin Invest.

[PDF_00348] Lichtiger S., Present D. H., Kornbluth A., Gelernt I., Bauer J., Galler G., Michelassi F., Hanauer S. (1994). Cyclosporine in severe ulcerative colitis refractory to steroid therapy.. N Engl J Med.

[PDF_00351] Munkholm P., Langholz E., Davidsen M., Binder V. (1994). Frequency of glucocorticoid resistance and dependency in Crohn's disease.. Gut.

[PDF_00367] O'Banion M. K., Winn V. D., Young D. A. (1992). cDNA cloning and functional activity of a glucocorticoid-regulated inflammatory cyclooxygenase.. Proc Natl Acad Sci U S A.

[PDF_00354] Pruimboom W. M., van Dijk J. A., Tak C. J., Garrelds I., Bonta I. L., Wilson P. J., Zijlstra F. J. (1994). Interactions between cytokines and eicosanoids: a study using human peritoneal macrophages.. Immunol Lett.

[PDF_00344] Schumert R., Towner J., Zipser R. D. (1988). Role of eicosanoids in human and experimental colitis.. Dig Dis Sci.

[PDF_00362] Shipman G. F., Bloomfield C. D., Gajl-Peczalska K. J., Munck A. U., Smith K. A. (1983). Glucocorticoids and lymphocytes. III. Effects of glucocorticoid administration on lymphocyte glucocorticoid receptors.. Blood.

